# Fascia as a regulatory system in health and disease

**DOI:** 10.3389/fneur.2024.1458385

**Published:** 2024-08-07

**Authors:** Alison M. Slater, S. Jade Barclay, Rouha M. S. Granfar, Rebecca L. Pratt

**Affiliations:** ^**1**^School of Population Health, The University of New South Wales, Sydney, NSW, Australia; ^2^Neuromuscular Imaging Research Lab, The Kolling Institute, St Leonards, NSW, Australia; ^3^Hypermobility and Performance Lab, Faculty of Medicine and Health, The University of Sydney, Sydney, NSW, Australia; ^4^School of Pharmacy and Medical Sciences, Griffith University, Southport, QLD, Australia; ^5^Department of Foundational Medical Studies, William Beaumont School of Medicine, Rochester, MI, United States

**Keywords:** fascia, connective tissue, collagen dysregulation, hypermobility, neuroregulation, mast cell, inflammation

## Abstract

Neurology and connective tissue are intimately interdependent systems and are critical in regulating many of the body’s systems. Unlocking their multifaceted relationship can transform clinical understanding of the mechanisms involved in multisystemic regulation and dysregulation. The fascial system is highly innervated and rich with blood vessels, lymphatics, and hormonal and neurotransmitter receptors. Given its ubiquity, fascia may serve as a “watchman,” receiving and processing information on whole body health. This paper reviews what constitutes fascia, why it is clinically important, and its contiguous and interdependent relationship with the nervous system. Unquestionably, fascial integrity is paramount to human locomotion, interaction with our environment, bodily sense, and general physical and emotional wellbeing, so an understanding of the fascial dysregulation that defines a range of pathological states, including hypermobility syndromes, autonomic dysregulation, mast cell activation, and acquired connective tissue disorders is critical in ensuring recognition, research, and appropriate management of these conditions, to the satisfaction of the patient as well as the treating practitioner.

## 1 Introduction

Despite being declared a global public health priority, chronic musculoskeletal pain and dysregulation conditions remain under-funded, under-researched, misdiagnosed and grossly misunderstood (1). Research and clinical paradigms that dismiss the importance of fascia in pain regulation can lead to undertreatment and inappropriate treatment for pathological states such as hypermobile Ehlers-Danlos Syndrome (hEDS), autonomic dysregulation, mast cell activation, and acquired connective tissue disorders. Continuing to ignore the fascial system perpetuates misdiagnoses and inappropriate treatments, creating unnecessary prescriptions of ineffective medication. Furthermore, application of incongruous allied health management protocols can cause masking of symptoms, and contributes to increases in physician burnout, preventable disabilities, and the global burden of chronic pain management in healthcare.

Teaching the form and function of fascia is notably absent from most professional health care education and the lack of awareness limits current treatments. Conventional healthcare has historically overlooked fascia, framing it not as a system that facilitates and modulates sensory input but as inert filler material (2). Understanding fascial anatomy and its multifaceted role as a regulatory system can transform how clinicians approach managing health and treating pain and disease.

By understanding the dynamics of the fascial system as it relates to the other organ systems of the body (musculoskeletal, neurovascular, endocrine, and immune)—we can illuminate its role in the regulation of pain, locomotion, inflammation, and autonomic conditions. People experiencing these conditions often have complex needs and difficult healthcare encounters, which can lead to clinical traumatization and mutual distrust between patients and clinicians (3). Continued research can help clinicians understand and improve care for “problem patients” by revealing an anatomical reason for their pain that travels and manifests in different ways on different days. This paper reviews the emerging body of evidence on fascia as a regulator of the body’s sensory input, and demonstrates how fascia-informed care can potentially improve patient outcomes and clinician experiences, ultimately preventing poor care across healthcare and medical specialties, particularly in the areas of neurovascular, hormonal, endocrine, musculoskeletal, inflammatory, and hypermobility-related conditions.

## 2 The regulatory role of fascia

### 2.1 Why fascia matters

Of the four major classes of tissues in the human body—endothelial, muscle, nerve, and connective tissue (fascia) (4)—fascia has historically been most overlooked and misunderstood in Western medicine (possibly due to ongoing nomenclature debates overshadowing clinically-relevant fascia studies (5). For the purpose of this review, we will use the terms *fascia* and *connective tissue* interchangeably). Previously thought to be a passive material, the cellular membrane is now widely known to be a dynamic structure critical to cellular functionality. Similarly, fascia, once thought to be inert material, has now been found to host membrane-like receptors and perform active membrane-like functions for structures it connects and encapsulates (6). Current research indicates that fascia could be the moderating interface between many tissue types in the musculoskeletal, endocrine, and autonomic nervous systems. Fascia surrounds, supports and protects every nerve, muscle, blood vessel, and organ in the body, and is abundantly innervated (7, 8). Fascia is estimated to house over 250 million nerve endings (8), with sensory neurons outnumbering motor neurons 9:1 in some regions (9). Fascia houses 25% more nerve endings than skin, and 1,000% more than the collective innervation of muscle, so fascia could very well be considered our richest sensory organ (7, 8).

Connective (fascial) tissue manifests in many forms, from the loose connective tissue immediately under the skin, to the deep connective tissue of the epimysium, perimysium and endomysium of muscle, to cartilage, tendon, ligament, to the protective wrapping around nerves and blood vessels, the periosteum and pericardium, and the visceral serous membrane. Traditionally discarded during anatomical dissection oriented toward muscles, organs, neurovascular bundles and bones, prominent clinicians and researchers note fascia’s emergence from obscurity as one of the most functionally diverse bodily components (2).

### 2.2 Functions of fascia

Fascia plays crucial roles in locomotion and regulation, and evolves in response to environmental stimuli and functional demand. Fascial integrity is paramount to movement, bodily sense, hormonal, autonomic and neurovascular regulation, and purposeful interaction with our environment. For movement and locomotion, fascia regulates posture (2), force transmission (10), strength generation (11), elastic recoil (12), proprioception (1), exteroception, and interoception (13). Fascia also regulates lymphatic efficacy (14), protection of delicate neural and vascular elements and organs (4), thermoregulation, inflammatory and immune responses (15), wound healing (16), hormonal production and secretion (adrenaline, estrogen, insulin, thyroid hormones, oxytocin) (17, 18), and venous return (19). Fascia plays a critical role in the transmission of neurotransmitters, namely serotonin, dopamine, GABA, and acetylcholine (17, 20). The regulation of peripheral resistance arteries by interfacing fascial tissue is essential for control of blood pressure, and for the increase in blood flow to the central nervous system and the heart under stress conditions (21). Alterations in the superficial fascia can also reciprocally lead to lymphedema/lipedema, which in turn may exacerbate suboptimal health of superficial fascia (22). Fascial morphology is initially determined by embryological and early childhood development (23), and undergoes perpetual adaptation across the lifespan in response to functional demand. Fascia is constantly evolving, both dependent on and modulating sustained postures, repetitive movement, quantity of motion, load (24), stress, strain, hydration (25), pH, temperature (23), neurotransmitters, and hormones (26).

### 2.3 Components of fascia

The fundamental components of fascial tissue are primarily specialized cells, collagen fibers, elastin fibers, and an extracellular matrix (ECM): (2).

- Cells provide the metabolic properties of the fascial tissue.
- Collagen and elastin fibers provide mechanical strength.
- ECM (also known as the ground substance) provides plasticity and elasticity.

Fascial cell types include fibroblasts, fasciacytes, adipocytes, macrophages and mast cells (27), undifferentiated mesenchyme cells, chondroblasts, chondrocytes, osteoblasts, and osteocytes (28). ECM contains hyaluronan (hyaluronic acid; HA), glycosaminoglycans (GAGs), water and ions (2). GAGs create an osmotic imbalance, which enables the ECM to attract up to 1,000 times its own weight in water (29). This hydrophilic quality of proteoglycans (glycoproteins that contain GAGs) is responsible for maintaining the volume of the extracellular matrix, which is constrained by the surrounding collagen fibers (30). The capacity for ECM to attract up to 1,000 times its weight in water is particularly important, because fascia is the “arena” in which localized acute inflammation and edema occurs (30).

Fascia evolves almost exclusively from the mesodermal layer during embryological development (23). Embryos first develop cerebrospinal fluid and fascia, then the remaining body systems and structures form within this “endless web” of fascia (23, 31). Fascia facilitates “a true continuity throughout our whole body” (2), and has been referred to as the “organ of form” (32), and “the architect of human movement” (22).

### 2.4 Functional demand specialization

Functional demand dictates the proportional distribution of each fascial component. Fascia develops perpetually across the lifespan in response to functional demand specific to each region of the body. Consider the specialization of three fascial components in response to specific functional demands:

- Type I collagen facilitates force transmission. For example, tendons and the thick aponeurotic sheets of the iliotibial band (33) and plantar fascia (34) facilitate force transmission (35, 36), so Type I collagen is the predominant fiber subtype.
- Fasciacytes facilitate sliding and gliding and secrete hyaluronan as a lubricant to facilitate movement. Where sliding and gliding is necessitated between muscle bellies, fasciacytes are distributed along the margins of the fascia that envelopes muscle (37), and hyaluronan is secreted to facilitate movement, particularly around the myofascial junction (38).
- Cartilage (also known as chondral tissue) is a highly specialized layer of dense ECM that allows almost frictionless motion in a joint. Cartilage is composed of hyaluronan, Type II collagen, and cartilage-specific proteoglycans interspersed sparsely with chondrocytes (39) enabling unique mechanical properties. Counterintuitively, “moderate mechanical loading” (40) on cartilage over time minimizes the turnover of tissue constituents, resulting in a protective effect rather than tissue degeneration (41).

### 2.5 Recognizing fascia dysregulation

Most clinical fascia literature focuses on temporary reversible impairment, assuming optimal homeostatic function in an otherwise healthy body. However, clinicians are often treating patients with (frequently undiagnosed) genetic variants altering the ECM fiber arrangement -- due to one or more changes affecting collagen proportions, quality, length, binding site affinities or distributions. In these cases, many of the “default” principles or “typical” assumptions do not apply, especially related to viscoelasticity, proprioception, nociception, elastic recoil and force transmission. When the usual ECM norms do not apply, the label “heterogenous” is often used to describe the diverse symptom presentations of individuals with conditions such as Hypermobile Ehlers-Danlos Syndrome (hEDS), Marfan Syndrome, acquired connective tissue disease including systemic lupus erythematosus (SLE), polyarthralgia, systemic sclerosis, and Sjogren Syndrome to name a few.

Multimorbidity is the norm among individuals with collagen dysregulation, with an average of more than 10 diagnosed conditions in patients with hEDS (42, 43). Commonly reported conditions include general joint hypermobility (subluxations and dislocations of joints, tendons, and nerves, frequent soft tissue injury, and abnormal wound healing) (42–46), tethered cord and cervical instability (47), a range of oral and orofacial manifestations (48), inflammatory bowel disease (49), dysautonomia, irritable bowel syndrome, reflux, diverticulosis (50), and postural orthostatic tachycardia syndrome (POTS) (51); cardiovascular disease including mitral valve prolapse and aortic wall hyperelasticity (52); dermal hyperextensibility and atrophic scarring (44), easy bruising (53), asthma (54), lipedema/lymphedema, chronic pain (55, 56), anesthetic resistance (57), fibromyalgia, myalgic encephalomyelitis or chronic fatigue syndrome (50), central sensitization (58), abdominal hernias and pelvic organ prolapses (44). Mast cell activation syndrome (MCAS), hypermobility and dysautonomia have been identified as a triad to be aware of in clinical presentation (59), with higher odds of autism, ADHD (60, 61), panic disorder (62), anxiety diagnoses (63, 64), and neurodivergence (56).

## 3 Fascia, exercise and locomotion

### 3.1 Hyaluronan: lubricant or glue?

Hyaluronan (hyaluronic acid) is the primary constituent of the ECM (65). Hyaluronan is secreted as a lubricant to facilitate myofascial sliding and gliding of muscles and nerves. Hyaluronan has been found to contribute to cellular metabolism (66), morphogenesis (67), wound healing (68), and inflammation (69). In local anesthetic studies, hyaluronan has been noted to effect the efficacy and release rate of anesthesia (70), which may explain local anesthesia resistance reported by patients with collagen dysregulation conditions (57). Dysregulated hyaluronan is associated with cancer development, and can result in excessive tissue swelling, increased interstitial pressure and compression of neurovascular structures, causing pressure and pain (66, 71).

Hyaluronan dysregulation can occur either through under-or over-activity (67). Under-activity can lead to hyaluronan over-accumulation; over-activity can lead to hyaluronan over-production. Immobility precipitates pathological accumulation of hyaluronan, which super-aggregates in the tissues, impairing blood and lymphatic circulation, and reducing lymphatic efficacy (68). Excessive exercise may also overstimulate hyaluronan production, and subsequent super-aggregation causes it to act more like a glue than a lubricant (67). When hyaluronan acts like a glue, it can lead to symptoms of lipedema/lymphedema including fat tissue inflammation, painful adipose tissue, adipose tissue growth, and fibrosis (69, 72). This phenomenon of hyaluronan acting more like a glue than a lubricant has also been linked to delayed onset muscle soreness syndrome (DOMS) (73).

### 3.2 Muscles, posture and movement

Immediately beneath the dermal layers (of skin), the superficial fascia transects two adipose layers (the superficial adipose tissue and deep adipose tissue). Superficial adipose tissue (SAT) regulates several aspects of whole-body physiology including insulin sensitivity, body temperature and immune responses (15). The adjacent superficial fascia plays a key role in the transmission of hormones and the protects invested vascular and neural plexuses (4). The superficial fascia is structured to support these circuitous structures, and facilitates the requisite flexibility to circumvent injury at the extremes of physiological range (74). The superficial fascia also includes the retinacula cutis, finger-like projections of collagen connecting the dermis above and hypodermis below, as well as the deep (muscular) fascia (75). The deep fascia comprises the epi-, peri-and endomysial layers of the muscular fascia. Collectively, these constitute the intramuscular connective tissue (IMT) (76), into which muscle fibers are wholly embedded (38, 77). With the seamless melding of the epi-and perimysial components, the IMT acts as a “scaffold” for muscle development and a carrier of the neural and vascular supply for muscle cells (76), in turn acting in series with the muscle itself and its associated mechanoreceptors. This allows forces generated within the locomotor system to be efficiently transmitted across joints (78), and so circumventing excessive articular strain.

Ruffini and Pacinian corpuscles are mechanoreceptors that appear in fascia in varying proportions (79). Ruffini corpuscles monitor persistent postural input, while Pacini receptors downregulate in response to continuous stimulus (80). “Myofascial expansions” (35)—wherein 30% of muscle fibers insert into fascia rather than a tendon (81)—facilitate force transmission (82) from both synergistic and antagonistic muscles into fascia rather than an enthesis. This accounts for 30% of the mechanical force (35, 83), helping to protect local neural and vascular tissue, as well as underlying joints (23), effectively allowing them to “float” (84). This calls into question some long-held anatomical paradigms around joint mechanics and load transfer in that muscle can no longer be considered the only element responsible for the organization of movement (35).

## 4 Fascia and regulation

### 4.1 Fascia and neuroregulation

The study of fascia demonstrates a specific distribution and precise localization of neural elements (8), closely connected with the central nervous system, and more so with the autonomic nervous system (ANS) (7). Local vasodilatation (80), and thermoregulation are primarily affected (85). In mast cell studies, fascia receptors have been found to respond to neurotransmitters more rapidly and at lower doses than neurons (86, 87). Treatments that target fascia regulation may have multiple uses. Current medical literature lacks fascia-focused research, and there is an identified need for studies designed specifically to evaluate the neuroregulation effects of treatments on fascia hyaluronan, and mast cells. Anxiety and depression have been associated with neuroinflammation, with mast cell stabilizers and treatments for inflammation yielding positive results (88). Limited clinical research has found ADHD medication to be effective in treating impulsivity and behavioral regulation in eating disorders (89), and has been reported in case studies to alleviate musculoskeletal and orofacial pain (90). The growing understanding of the fundamental neural dynamics of fascial plasticity has catalyzed a paradigm shift in the manual therapy approach to treatment of fascial dysfunction, moving away from exclusive consideration of fascia’s mechanical properties, and calling into question the categorization of massage, tape, and compression as “passive therapies” (2). Chemical alterations of the extracellular matrix, and excessive mechanical stimulation are known to transform these receptors into nociceptors, which are appreciably more sensitive than the underlying musculature, sustaining longer-lasting hypersensitivity (79).

### 4.2 Inflammation, stress, anesthesia and pain

The dominance of sympathetic innervation has far-reaching consequences for fascial health and aspects of the hypothalamic–pituitary–adrenal (HPA) axis, given the abundance of autonomic neural input in this tissue (91, 92). Chronic stress and chronic pain are—jointly and individually—often accompanied by chronic inflammation (93, 94), with pro-inflammatory cytokines secreted by fibroblasts, myofibroblasts, adipocytes, mast cells, lymphocytes and vascular cells. As the fascia is the principal setting for inflammatory and immune system activity, fascial dysfunction can result in catastrophic cascades (79, 95, 96). Even under conditions of relatively low-grade chronic inflammation, cytokines denigrate the ECM, destabilizing connective tissue and stimulating fibrosis (87). Noradrenaline upregulates Transforming growth factor–β1 (TGF-β1), which can cause fibroblasts to differentiate into myofibroblasts, which (in an environment of chronic stress and/or inflammation) then initiates fibrosis and contractures (97). Chronic adrenaline upregulation can similarly elicit contracture and inhibit effective wound healing (98), provoking structural adaptation. This in turn creates imbalance, pain and palpable tension (98–100). Stress hormones, like cortisol, and chronic inflammation can exert an adverse effect on muscle and bone quality (101, 102). The gut, with its enteric nervous system (little brain), is compartmentalized by fascia (103) and produces the same neurotransmitters as the brain. The vagus nerve purportedly acts as a bi-directional autonomic conduit between fascia and the central nervous system in countering sympathetic overload, as well as playing a key role in the gut-brain axis, tempering inflammation, facilitating intestinal homeostasis, satiety and energy regulation (104). The vagus nerve also connects the gut and the brainstem (36, 104), further strengthening the close relationship between fascial and the nervous systems.

Fascia appears to act as a mediator between the autonomic nervous system, emotional regulation and immune regulation (79). An emerging body of research has begun to explore the dynamic interplay between fascia and cancer, fascia and muscle function, fascia and neuroinflammation, dysautonomia, pain, hypermobility, and neurodiversity (56, 71, 76, 105), with some studies focused on the interaction of fascia, neurotransmitters, hormones, and the hypothalamic–pituitary–adrenal axis (HPA axis) (95, 98). Additionally, emerging research posits that fascia and hyaluronan play a key role in the perception of pain (1, 66, 106). Fascia is also richly endowed with endocannabinoid receptors, signifying its role as a source and modulator of pain, presenting possibilities for future pain management strategies and research (107).

While fascia can make inflammation and pain worse, fascial regulation of inflammation can also provide an efficient therapeutic target to counteract chronic pain-inflammation-stress cascades. While MCAS is rarely the first diagnosis received (it is often the last of many), simple treatments targeting mast cell stabilization or inflammatory regulation in fascia like olopatadine (in Patanol eye drops or Patanase and Ryaltris nasal sprays), or sodium cromoglycate (also known as cromolyn sodium) have been reported to rapidly reduce these cascades, with topical or nasal application (86, 108–110).

Several drugs are associated with mast cell activation, including nonsteroidal anti-inflammatory drugs (NSAIDs), anesthetics, neuromuscular blocking agents and opiates/opioids, and contrasts used in radiology (87, 111). Approximately half of all perioperative drugs can trigger mast cell activation (111), and hyaluronan affects anesthesia efficacy and release rate (70), necessitating research into hyaluronan and mast cell receptors in connective tissue, and substances that bind with these receptors (or silence receptor-signaling) as therapeutic targets. One such receptor, Mas-related G protein-coupled receptor-X2 (MRGPRX2), binds with opioids and naltrexone, and has been associated with fibrosis, hypertrophic scarring, and Long COVID (87, 111). Low dose naltrexone (LDN) is thought to bind with these mast cell receptors, and research has begun to investigate its potential to reduce side effects of immunosuppressive therapies (86, 88, 110), enhance analgesia, reduce proinflammatory mediators, and efficacy as a treatment for MCAS, anxiety and depression, and neuropathic pain (110). After successful therapeutic responses in studies with back pain, plantar fasciitis and Long COVID (106, 112, 113), intravenous (IV) saline and IV immunoglobulin (IVIG) have been hypothesized to treat underlying fascia-immune dysregulation; which goes underrecognized and undertreated in a wide range of health conditions.

### 4.3 Mast cells and neuro-immuno-endocrine regulation

Mast cells (also known as mastocytes) are tissue-resident granulocytes of myeloid lineage that play a central role in adaptive and innate immune function, neurological and non-immunological processes, and pathologies far beyond allergy and mastocytosis. Mast cells—dubbed a “multi-functional master cell” (114)—are found in connective tissue, vascular tissue, adipose tissue, and lymphatic tissue around the body, and are highly concentrated and recruited to junctions where antigens could enter the body, including all mucosal openings, skin, blood, respiratory endothelium, and gastrointestinal tract (27, 114). In response to environmental stimuli, mast cell activation and degranulation modulates vasodilation, blood pressure, nociception, itch, fibrosis, tissue permeability, wound healing, inflammation and immune responses, and behavior (27, 114–116). Two distinct mast cell phenotypes have been identified, distinguished by granule content. Unlike mucosal mast cell granules that predominantly contain tryptase, mast cell granules of non-mucosal tissue mastocytes contain a wide range of specialized enzymes (114, 116, 117), and are rich pharmacological targets (118). When activated, mast cells release the content of the granules, which can have major local and systemic effects, particularly if mast cell derived mediators circulate via the vascular or lymphatic systems (86, 110).

Mast cells can also be activated in response to environmental changes, including temperature, pressure, and dermal vibration (119, 120). Mast cell activation contributes to swelling, itch, fasciitis, and predisposes an environment toward fibrosis via various mechanisms including fibroblast recruitment and proliferation, hyaluronan degradation, or excessive ECM deposition. With disruption or underlying dysregulation of connective tissue, a normal regulatory response may lead to a pathological outcome. When mast cells release enzymes and mediators (including histamine, cytokines, and hundreds more) (38, 116) in the neuromuscular-myofascial junctions, it may evoke an escalating cascade of multisystemic dysregulation and localized deterioration. It is worth noting that MCAS and mast cell activation disease (MCAD) are not interchangeable terms (121). The term MCAD includes several subtypes including systemic mastocytosis which is characterized by excessive quantities of mast cells, and MCAS which involves the inappropriate release/levels of mast cell chemicals. While the symptom presentation of these conditions is similar, the different pathomechanisms indicate different treatment plans. As mast cell activation increasingly features in cases of chronic pain-inflammation-stress cascades and anesthesia resistance within hypermobile populations and those with autonomic dysregulation (57, 59, 122), treatment plans that prioritize mast cell inhibition could prevent or reduce these cascades and increase the efficacy of multidisciplinary treatment protocols. This adds to the regulatory explanation for medication sensitivity and paradoxical responses to medications, and presents a strong case to target mast cells in clinical research and therapeutic intervention.

### 4.4 Hormonal regulation in muscular fascia

Research has discovered an abundance of estrogen and relaxin receptors in fascia, concentrated on the fibroblasts of muscular fascia (26). Hormone concentrations are known to fluctuate significantly throughout the menstrual cycle and pregnancy, declining with the onset of perimenopause (123). Hormones directly influence fascial stiffness and pain sensitization throughout the lifespan of people who menstruate, due to hormonal inhibition of fibrosis and inflammation resulting in ECM remodeling (25). This has major consequences in the consideration of athletes at any age, especially those with a hyper-flexible fascial system, with or without the presence of joint hypermobility, and with or without the use of hormone therapies or oral contraceptives (124). People experiencing perimenopause and menopause may subsequently be more susceptible to fibromyalgia in the absence of premenopausal hormone concentrations (125).

## 5 Conclusion

### 5.1 Implications for practice and research

Dysregulation within the fascial system is considered scientifically complex and likely plays a pivotal role in the multimorbidity encountered by patients living with connective tissue diseases (diagnosed or not). Various stages of fascial health may lead to paradoxical or atypical responses to “normal” manual therapy, or pharmacotherapy; making walking or exercise painful or otherwise unmanageable by the patient. When therapy causes pain, fascial impairment should be strongly considered and explored so treatment plans can better address the culprit of chronic pain. We call for clinical educators and researchers, and the next generation of healthcare practitioners to normalize the understanding of hyaluronan dysregulation and the fascial system and their ubiquitous role in whole body health, diagnosis and treatment planning, inflammation and pain management, and homeostasis.

## Author contributions

AS: Conceptualization, Writing – original draft, Writing – review & editing. JB: Conceptualization, Writing – original draft, Writing – review & editing. RG: Writing – review & editing. RP: Writing – original draft, Writing – review & editing.
